# The Pro-neurogenic Effects of Cannabidiol and Its Potential Therapeutic Implications in Psychiatric Disorders

**DOI:** 10.3389/fnbeh.2020.00109

**Published:** 2020-06-26

**Authors:** Miguel Á. Luján, Olga Valverde

**Affiliations:** ^1^Neurobiology of Behaviour Research Group (GReNeC—NeuroBio), Department of Experimental and Health Sciences, Universitat Pompeu Fabra, Barcelona, Spain; ^2^Neuroscience Research Programme, IMIM-Hospital del Mar Research Institute, Barcelona, Spain

**Keywords:** cannabidiol, drug addiction, substance use disorder, endocannabinoid system, neurogenesis, hippocampus

## Abstract

During the last decades, researchers have investigated the functional relevance of adult hippocampal neurogenesis in normal brain function as well as in the pathogenesis of diverse psychiatric conditions. Although the underlying mechanisms of newborn neuron differentiation and circuit integration have yet to be fully elucidated, considerable evidence suggests that the endocannabinoid system plays a pivotal role throughout the processes of adult neurogenesis. Thus, synthetic, and natural cannabinoid compounds targeting the endocannabinoid system have been utilized to modulate the proliferation and survival of neural progenitor cells and immature neurons. Cannabidiol (CBD), a constituent of the *Cannabis Sativa* plant, interacts with the endocannabinoid system by inhibiting fatty acid amide hydrolase (FAAH) activity (the rate-limiting enzyme for anandamide hydrolysis), allosterically modulating CB1 and CB2 receptors, and activating components of the “extended endocannabinoid system.” Congruently, CBD has shown prominent pro-neurogenic effects, and, unlike Δ^9^-tetrahydrocannabinol, it has the advantage of being devoid of psychotomimetic effects. Here, we first review pre-clinical studies supporting the facilitating effects of CBD on adult hippocampal neurogenesis and available data disclosing cannabinoid mechanisms by which CBD can induce neural proliferation and differentiation. We then review the respective implications for its neuroprotective, anxiolytic, anti-depressant, and anti-reward actions. In conclusion, accumulating evidence reveals that, in rodents, adult neurogenesis is key to understand the behavioral manifestation of symptomatology related to different mental disorders. Hence, understanding how CBD promotes adult neurogenesis in rodents could shed light upon translational therapeutic strategies aimed to ameliorate psychiatric symptomatology dependent on hippocampal function in humans.

## Introduction

Neuropsychiatric disorders such as schizophrenia, mood disorders, or drug addiction, represent a huge burden on society, greatly impairing the health of those affected. During the last half-century, considerable progress has been made to understand, prevent, and treat such conditions. However, treatment options are still far from optimal in terms of efficacy and specificity, and there remain important untreatable maladaptive phenotypes and treatment-resistant patients. To solve this issue, basic and applied research has tried to identify new altered neuropsychological mechanisms suitable to promote new therapeutic strategies (Cuthbert, 2014). In this quest, the discovery of adult hippocampal neurogenesis (Altman and Das, 1965) and its health implications (Kempermann, 2012) has opened new vistas upon innovative pharmacotherapies that could ameliorate impaired hippocampal function. Among the many ways proposed to accomplish such an improvement, cannabidiol (CBD) has recently stood out as a promising compound to be taken into consideration. In light of this, the following mini-review article aims to: (1) summarize the available evidence describing the modulation of adult hippocampal neurogenesis by CBD; to (2) provide a prospective collection of the responsible mechanisms; and (3) to detail the presumed therapeutic potential of this phytocannabinoid *via* the modulation of adult neurogenesis.

### Cannabidiol

CBD is one of the most abundant constituents of the *Cannabis sativa* plant. Unlike Δ^9^-tetrahydrocannabinol (THC), CBD is devoid of psychotomimetic and rewarding effects (Ligresti et al., 2016), and is well tolerated in humans (Chesney et al., 2020). CBD is thought to interact with several molecular targets (Campos et al., 2017). Its main targets within the central nervous system are comprehended by the activation of 5-hydroxytryptamine 1A (5-HT_1A_), transient potential vanilloid 1 (TRPV1), G-protein 55 (GPR55) and peroxisome proliferator-activated gamma (PPARγ) receptors, as well as the antagonism of adenosine reuptake (Turner et al., 2017). Despite initial controversy about its endocannabinoid targets (Zlebnik and Cheer, 2016), recent evidence also supports CBD as a negative allosteric modulator of cannabinoid receptors 1 and 2 (CB1, CB2) at physiologically relevant concentrations (Laprairie et al., 2015; McPartland et al., 2015; Martínez-Pinilla et al., 2017; Navarro et al., 2018; Tham et al., 2019). Also, CBD reduces anandamide (AEA) metabolism by inhibiting fatty acid amide hydrolase (FAAH) activity (De Petrocellis et al., 2011). Consequently, CBD is an efficient anxiolytic (Fogaça et al., 2018) and there is evidence suggesting that it possesses anti-inflammatory (Atalay et al., 2019), neuroprotective (Campos et al., 2016), antidepressant (Sales et al., 2019), anti-relapse (Gonzalez-Cuevas et al., 2018), pro-cognitive (Osborne et al., 2017) and antipsychotic (Renard et al., 2017) effects. Accordingly, CBD has been proposed as a novel therapeutic strategy for different mental disorders such as drug addiction (Calpe-López et al., 2019), depression (Silote et al., 2019), or schizophrenia (Elsaid and Le Foll, 2020). Notwithstanding the foregoing, CBD has a formidably complex pharmacology, and therefore, we lack a clear understanding of the molecular and neuroplastic consequences of CBD treatments. With such a pool of targets, numerous hypotheses have tried to explain CBD’s therapeutic mechanisms in each of the psychiatric models addressed. The modulation of neuronal network dynamics in the mesolimbic system *via* 5-HT_1A_ activation (Norris et al., 2016) is positioned as the best approximation to CBD’s anti-craving actions (Katsidoni et al., 2013; Bi et al., 2019; Galaj et al., 2020). On the other hand, the presumed motivational consequences of *in vivo* CBD’s CB1 effects remain unclear. Recent reports show that CBD modulation of cocaine-seeking reinstatement, but not operant intake, depends on CB1 receptor activation (Galaj et al., 2020; Lujan et al., 2020). Therefore, indirect CB1 activation through FAAH blockade, rather than CB1 negative allosteric modulation, is a more plausible mechanism for the anti-craving effects of CBD. In the case of mood and anxiety-related disease models, the activation of ventromedial prefrontal cortex 5-HT_1A_ and CB1 receptors (Linge et al., 2016; Sartim et al., 2016), and the neuroprotection against inflammatory and oxidative brain insults (Campos et al., 2016) are the main mechanism candidates. Lastly, diverse studies have also pointed to the pro-neurogenic effects of CBD, as reviewed below.

### Adult Hippocampal Neurogenesis in the Mammalian Brain

**Figure 1:**
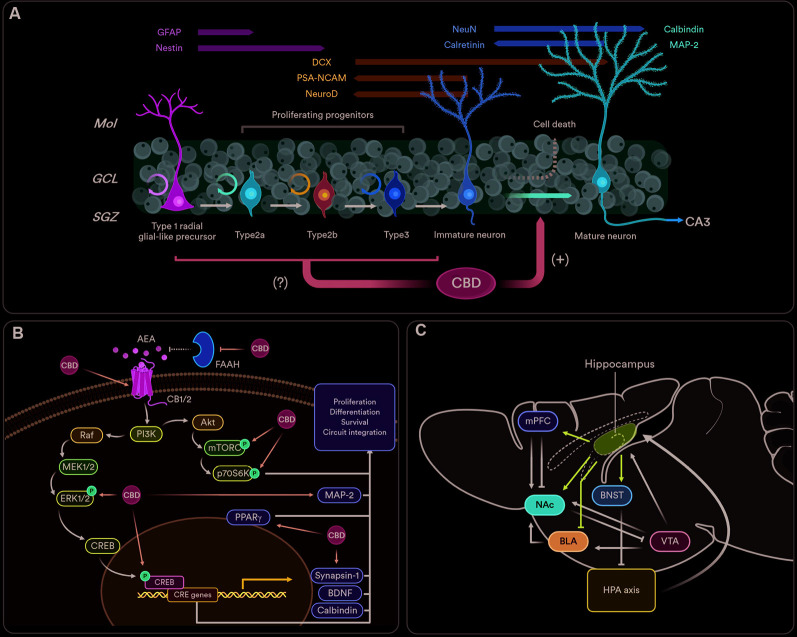
The pro-neurogenic effects of cannabidiol (CBD) and its functional relevance. **(A)** Adult hippocampal neurogenesis originates from type 1 precursor cells that might differentiate into granule neurons (Kriegstein and Alvarez-Buylla, 2009). The newly generated cells can be stimulated *via* GABAergic, endocannabinoid- and serotonin-dependent mechanisms (Encinas et al., 2006; Prenderville et al., 2015). The proliferation phase ends just after precursor cells exit the cell cycle. As early as 1 day after such an event, newborn neurons start expressing the postmitotic marker NeuN, which then declines as most newborn cells are eliminated before they become functional (survival phase; Kempermann et al., 2015). Within days after generation, newborn neurons send their axons to cornu ammonis 3 (CA3), where they form proper synapses (Sun et al., 2013). In the next phase, newborn neurons mature as the excitatory nature of GABA inputs shift into the standard depolarizing profile. Finally, new mature neurons go through a phase of increased synaptic plasticity, which in turn promotes its final integration into the hippocampal circuitry (functional integration phase; Ge et al., 2007). The effects of CBD are preferentially focused on the post-mitotic stages of the neurogenic process, whenever it facilitates neuronal maturation and impedes early neuronal death.** (B)** The pro-neurogenic effects of CBD are orchestrated by the eCB system. Furthermore, CBD upregulates different molecular components of downstream pathways usually associated with the eCB-driven facilitation of adult neurogenesis. Final molecular effectors of the protein synthesis and survival machinery of the hippocampus such as brain-derived neurotrophic factor (BDNF), calbindin, MAP-2, synapsin 1, and the activation of protective peroxisome proliferator-activated gamma (PPARγ) receptors, are also found upregulated after CBD subchronic treatments. **(C)** The figure represents a simplified vision of the hippocampal neurocircuitry functionally coupled to the neurogenic state of the dentate gyrus (DG). The hippocampus (ventral part) sends direct projections to the GABAergic interneurons of the BNST that, in turn, tune-down the hypothalamus–pituitary–adrenal (HPA) axis (Snyder et al., 2011). Direct projections from the hippocampus to the mPFC promote stress sensitivity (Padilla-Coreano et al., 2016), and mediate antidepressant effects (Bagot et al., 2015). Ventral hippocampal outputs to the BLA are involved in the feedforward inhibition of fear and anxiety-related responses (Bazelot et al., 2015). Finally, the hippocampus can indirectly influence VTA DA release in motivated tasks by activating medium spiny neurons of the NAc (Britt et al., 2012). Abbreviations: GFAP, glial fibrillary acidic protein; Mol, molecular layer; GCL, granule cell layer; AEA, anandamide; mPFC, medial prefrontal cortex; NAc, nucleus accumbens; BLA, basolateral amygdala; BNST, bed nucleus of the stria terminalis; VTA, ventral tegmental area. Created with Biorender.com.

Adult hippocampal neurogenesis encompasses a complex, multistep process comprehending the proliferation, survival, differentiation/maturation, and functional integration of newborn neurons residing in the subgranular zone (SGZ) of the dentate gyrus (DG; Kuhn et al., 2018; Figure 1A). It is detailed in most mammals (Amrein, 2015), but its existence in humans has been hotly debated due to the critical dependence on ^14^C labeling (Sorrells et al., 2018). However, the latest evidence suggests that adult hippocampal neurogenesis in humans is abundant even in the senescence (Boldrini et al., 2018; Tobin et al., 2019) and that previous discrepancies were probably due to tissue processing protocols or neurological illness of the tissue donors (Moreno-Jiménez et al., 2019).

During their development, adult-born neurons modulate DG functions that orchestrate diverse behaviors. Newborn neurons act as independent encoding units that can inhibit the activity of mature granule cells (Drew et al., 2016) and dampen overall DG excitability (Ikrar et al., 2013). Given the participation of hippocampal function in mood, cognition, and motivation, adult hippocampal neurogenesis is involved in different neuropsychological processes in physiological and pathological conditions (Mandyam and Koob, 2012). For example, patients with depression exhibit decreased levels of neurogenesis (Lucassen et al., 2010). Neurogenesis ablation increases innate anxiety-like behaviors (Revest et al., 2009) and depressive-like symptoms (Wu et al., 2014) in animal models. And more importantly, anti-depressant drugs increase neurogenesis, an effect that is required to observe some of its behavioral effects in rodents (Santarelli et al., 2003). Such relations are thought to be due to the newborn neuron regulation of hippocampal inhibitory control over the hypothalamus–pituitary–adrenal (HPA) axis. The ventral part of the hippocampus has also been related to emotional control. This region shares regulatory projections to canonical emotional-processing structures such as the basolateral amygdala or the medial prefrontal cortex that are key to modulate fear-associated memories and anxiety (Felix-Ortiz et al., 2013; Padilla-Coreano et al., 2016) and are regulated by neurogenesis (Temprana et al., 2015; Figure 1C). Interestingly, it is now described that pattern separation, a brain computational mechanism dependent on newborn neurons (Leutgeb et al., 2007; Sahay et al., 2011) also allows for the discrimination of emotional states experienced during memory creation (Redondo et al., 2014), thus granting adult hippocampal neurogenesis a way to modulate emotional memories retrieval and storage (Anacker and Hen, 2017). There is debate as to whether such functional implication could represent a caveat of pro-neurogenic therapeutics, as enhanced pattern separation processes may promote proactive interference (see Epp et al., 2016; Tello-Ramos et al., 2019).

Additionally, the circuitry involved in the regulation of mood and stress overlaps with the brain circuitry affected by motivation disorders (Koob, 2015; Volkow et al., 2016). Hence, extensive evidence posits adult hippocampal neurogenesis as an additional component of drug addiction etiology (Castilla-Ortega et al., 2016; Barr et al., 2018). In this sense, rats with experimentally-reduced neurogenesis consume more cocaine and work harder to obtain the drug (Noonan et al., 2010; Deroche-Gamonet et al., 2019). Conversely, pharmacological induction of adult neurogenesis facilitates the forgetting of cocaine-contextual memories (Ladrón de Guevara-Miranda et al., 2019). Other drugs, such as alcohol, induce persistent reductions in adult neurogenesis in rodents (Spear, 2018), primates (Taffe et al., 2010), and humans, as shown by *post mortem* samples from alcohol abusers (Dhanabalan et al., 2018). Finally, the neonatal ventral hippocampal lesion rat model, which irreversibly lessens adult neurogenesis, has been used to reveal the participation of adult neurogenesis in the pathogenesis of dual diagnosis schizophrenia (Chambers and Self, 2002). This relation suggests that neurogenic deficits may also underlie positive-like, negative, and cognitive symptoms of schizophrenia in rodent models (Chambers, 2013; Sentir et al., 2020). Overall, a wealth of literature supports the relevance of adult neurogenesis in preclinical models of mood and anxiety disorders, as well as drug addiction or schizophrenia, while opens a new window of therapeutic opportunities aimed to ameliorate impaired hippocampal function.

## Promoting Neurogenesis with Cannabidiol

### Preclinical Evidence

Considering that the endocannabinoid (eCB) system exerts important functions in the regulation of neuronal generation and survival (Aguado et al., 2005), the Kempermann’s group firstly explored the possibility that a cannabinoid like CBD could enhance the survival of DG newborn neurons in mice (Wolf et al., 2010). The pioneering study showed that a CBD-enriched diet increased co-localized immunoreactivity of 5-bromo-2′-deoxyuridine (BrdU) and *neuronal nuclei* (NeuN). Moreover, the authors reported an interesting opposition to the effects of THC on this measure. Months later, Demirakca et al. (2010) similarly proposed these pro-neurogenic actions of CBD in humans. Since then, researchers have echoed these investigations, finding a remarkable result consistency in the pro-neurogenesis induced by CBD (Table 1).

**Table 1:** Literature assessing the effects of cannabidiol (CBD) in adult hippocampal neurogenesis.

Reference	Animal, cell line	CBD treatment protocol	Markers	Effect	Experimental condition
Wolf et al. (2010)	C57BL/6 female mice	CBD-enriched diet, 6 weeks	BrdU/Nestin/DCX^–^ (early proliferation)	–	–
			BrdU/Nestin/DCX^+^ (late proliferation)	↓	–
			BrdU/NeuN (late survival)	↑	–
Esposito et al. (2011)	Sprague–Dawley male rats	10 mg/kg, i.p., 15 days	DCX	↑	Aβ-inoculated rats
Campos et al. (2013)	C57BL/6 male mice	30 mg/kg, i.p., 14 days	DCX	↑	Naive, chronic unpredictable stress
			BrdU/NeuN	↑	Control	
	Hippocampal HiB5 progenitors	50, 100, 250, 500 nM, 18 h	BrdU/NeuN (proliferation)	100, 250 mg/kg: ↑	–
				50, 500 mg/kg:–
Shinjyo and Di Marzo (2013)	Mouse neural stem/progenitor cells	1 μM, 2 days	Nestin	↑	–
Schiavon et al. (2016)	Swiss CD-1 male mice	3 and 30 mg/kg, i.p., 15 days	DCX (proliferation)	3 mg/kg: ↑	–
				30 mg/kg: ↓
Mori et al. (2017)	C57BL/6 male mice	10 mg/kg, i.p., 3 days	DCX	↑	Ischemic mice
			MAP-2 (dendritic maturation)	↑	Ischemic mice
Fogaça et al. (2018)	C57BL/6 male mice	30 mg/kg, i.p., 14 days	DCX	–	Control
				↑	Chronic unpredictable stress
			DCX-tagged cell migration	–	Control
				↑	Chronic unpredictable stress
			BrdU/NeuN	–	Control
				↑	Chronic unpredictable stress
Luján et al. (2018)	Swiss CD-1 male mice	20 mg/kg, i.p., 10 days	BrdU/NeuN	↑	Control and cocaine-consuming mice
			DCX	↑	Cocaine-consuming mice
Luján et al. (2019)	Swiss CD-1 male mice	10, 20 mg/kg, i.p., 10 days	BrdU/NeuN	↑	Control and cocaine-consuming mice
			DCX	↑	Control and cocaine-consuming mice
Bis-Humbert et al. (2020)	Sprague-Dawley male rats	3, 10, 30 mg/kg, i.p., 6 days	NeuroD	–	–

*In vivo measures of BrdU incorporation or Ki67 not accompanied by a neuronal marker (e.g., NeuN) were not considered, given the difficulty to differentiate from the proliferation of non-neuronal cellular lineages. Abbreviations: BrdU, 5-bromo-2′-deoxyuridine; NeuN, neuronal nuclei; DCX, doublecortin; MAP-2, microtubule-associated protein 2. –, no change found; ↑, increase; ↓, decrease*.

Much of the preclinical work aimed at delineating the pro-neurogenic profile of CBD has mainly utilized two immunostaining observables: doublecortin (DCX) and BrdU/NeuN. Due to the prolonged presence of both markers in different stages of the neurogenesis process (for a review see Kempermann et al., 2015), it is difficult to elucidate the phase specificity of CBD changes. CBD increased BrdU/NeuN co-localization from 1 month after the injection of the thymine incorporation tracer (Wolf et al., 2010; Fogaça et al., 2018), a measure of late survival, to as early as 7 days (Luján et al., 2018, 2019), a correlate of early differentiation. The same consistency has been found using DCX. Following the same treatment protocol, CBD increased DCX staining from 7 days (Luján et al., 2019) to 1 month (Luján et al., 2018) after the last CBD injection. But in the work of Wolf et al. (2010), CBD did not enhance, and even reduced, neural progenitor cell (BrdU/Nestin-expressing type 1/2 cells) proliferation. More studies analyzing markers of neural progenitor cell proliferation are needed but, these results could imply that CBD pro-neurogenic effects would take place after newborn neurons are generated, and not before (Figure 1B). This goes in agreement with molecular findings reflecting the facilitating effects of CBD on postmitotic neuronal survival, differentiation, and maturation. The brain-derived neurotrophic factor (BDNF) positively regulates newborn neuron survival in the DG (Waterhouse et al., 2012), and CBD increases BDNF protein content within the hippocampus (Mori et al., 2017; Luján et al., 2018; Sartim et al., 2018; Sales et al., 2019). Calbindin, a Ca^2+^-binding protein used as a marker of mature neurons (Brandt et al., 2003), is also increased in the hippocampus of CBD-treated rats (Esposito et al., 2011). This idea has been further corroborated by the discovery that CBD activates different survival and synaptic remodeling cascades such as ERK1/2-CREB (Luján et al., 2018), GSK3β and PSD95 (Campos et al., 2013) or PI3K/mTOR/p70S6K (Renard et al., 2016; Giacoppo et al., 2017; Lanza Cariccio et al., 2018).

CBD pro-neurogenesis also shows great consistency across doses. Literature findings report increases in neuronal proliferation and differentiation after CBD doses ranging from 3 to 30 mg/kg, usually after prolonged treatments (≥10 days; Table 1). Despite this, at least two studies point to an inverted U-shaped dose-response curve effect. *in vitro*, Campos et al. (2013) described that CBD enhanced neuronal proliferation at medium concentrations (100, 250 nM), but these effects disappeared at lower (50 nM) or higher doses (500 nM). Similarly, Schiavon et al. (2016) showed that neuronal proliferation enhancement (here assessed by DCX) could only be observed after low (3 mg/kg) but not high (30 mg/kg) doses *in vivo*. Inverted U-shaped dose-response curves usually suggest the participation of multiple pharmacological mechanisms. In this way, it has already been described that CBD also exerts a similar anxiolytic dose-response curve (for a review see Jurkus et al., 2016) and that it is due to the interaction of 5-HT_1A_ and TRPV1 mechanisms (Campos and Guimarães, 2009). Therefore, one of the first neurogenic mechanisms that were evaluated consisted of the activation of 5-HT_1A_ receptors. However, CBD-induced proliferation in HiB5 hippocampal progenitor cells was not blocked by a 5-HT_1A_ antagonist (Campos et al., 2013) and so, an alternative candidate was considered: the eCB system.

### Evaluating CBD’s Endocannabinoid Mechanisms to Promote Neurogenesis

The eCB system stands out as a key regulator of newborn neuron generation, survival, maturation, and functional integration in the adult hippocampus. Neural progenitor cells, and their descendants, express a functional eCB system and are subject to the effects of endocannabinoid signaling (Prenderville et al., 2015). CB1 agonists induce neural proliferation and differentiation in the DG (Andres-Mach et al., 2017), which are also attenuated in CB1^−/−^ mice (Aguado et al., 2007; Zimmermann et al., 2016). The same has been detailed for CB2 receptors (Palazuelos et al., 2012; Avraham et al., 2014), although in a more complicated fashion (Rodrigues et al., 2017; Mensching et al., 2019). That is, CB1 receptors participate in the maintenance of adult neurogenesis, whereas CB2 receptors seem to promote the recovery from allostatic neurogenic states (Oddi et al., 2020). Furthermore, the intricate downstream cellular pathways engaged by cannabinoid receptors, mainly converging in Akt/mTORC and MAPK/CREB pathways, are critically involved in cell proliferation, differentiation, and survival and are required for endocannabinoids to exert its pro-neurogenic effects (Prenderville et al., 2015).

Given the mechanistic interactions between CBD and eCB system, a plausible hypothesis originated stating that CBD increases adult neurogenesis by modulating the eCB system. Accordingly, *in vitro* and *in vivo* evidence has suggested such interplay. The first evidence was given by Wolf et al. (2010). In their study, a CBD-enriched diet facilitated newborn neuron survival, an effect prevented in CB1^−/−^ mice. The seminal work of Campos et al. (2013) further explored this idea and tested which molecular mechanisms could be involved *in vitro*. CB1 and CB2 antagonists prevented the pro-neurogenic effect of CBD in hippocampal HiB5 progenitor cells. Furthermore, CB1 and CB2 receptor agonists, as well as eCB degradation inhibitors mimicked the pro-neurogenic effects of CBD. Interestingly, CBD effects were abrogated when the FAAH was inhibited. Combined, these results imply that the pro-neurogenic effects of CBD depending on the increase of AEA concentration. Crucially, CBD is an inhibitor of the FAAH and is well known to increase AEA concentration (Bisogno et al., 2001; De Petrocellis et al., 2011; Leweke et al., 2012; Petrosino et al., 2018). Note that, in this case, the CBD-induced negative allosteric modulation of CB1 receptors should not account for these results, as they rely on the facilitation of CB1 function. Alternatively, CBD can also increase the protein content of CB1 receptors in the hippocampus (Luján et al., 2018). Recently, a similar mechanism was revealed *in vivo*. After a CBD treatment in chronically stressed mice, neuronal differentiation, and late survival were found to be increased in CBD-treated mice (Fogaça et al., 2018). Such pro-neurogenic effects depended on CB1 and CB2 receptor activation, insofar respective antagonists abolished said increase. Intriguingly, CB1 antagonism only prevented the DCX-labeled neuronal differentiation increase whereas the CB2 antagonist precluded the increment of both, neuronal differentiation and late survival (BrdU/NeuN; Fogaça et al., 2018). Regarding this divergence, previous works have indicated that CB1 receptors may be implicated in maintaining basal adult neurogenesis, while CB2 receptors might be more physiologically relevant in coping with neurotoxic brain insults (Oddi et al., 2020). In the study of Fogaça et al. (2018), possibly the CB2 outshined CB1 receptors because its relative contribution was exclusively performed in chronically-stressed mice. So far, the differential role of CB1 and CB2 receptors in the pro-neurogenic effects of CBD in normal and allosteric conditions has not been explored enough, and more studies are needed to address this question. Altogether, studies interrogating the eCB system in conditions in which CBD produced pro-neurogenic effects have all encountered a suggesting implication. Although promising, there remain important gaps to be filled. For instance, no data is available as to the eCB-specific downstream signaling pathways recruited by cannabinoid receptors that would be facilitating neuronal survival and differentiation, despite some approximations in this regard (Luján et al., 2018). Furthermore, there also remain some unexplored CBD mechanisms with potential pro-neurogenic properties, such as GPR55 activation for coping reduction of neurogenesis in response to inflammatory insults (Hill et al., 2019). Noteworthy, a protective interaction involving neuroinflammation processes has been already observed, showing that CBD-mediated activation of PPARγ is associated with increased neurogenic activity, as well as reduced reactive gliosis, in the granule cell layer of the hippocampal DG (Esposito et al., 2011).

## Therapeutic Insights From Preclinical Psychiatric Models

A considerable number of studies have reported the pro-neurogenic effects of CBD, and some among them have even related these with an eCB mechanism of action. But, can the pro-neurogenic effects of CBD account for some of its therapeutic applications? Answering this question requires specialized experimental strategies designed to rule out CBD pro-neurogenesis, leaving intact its other pharmacological mechanisms and so, fewer experiments have been conducted. Nonetheless, a handful of studies have addressed this question, presenting evidence for a potential implication in the protection against neurodegenerative diseases (Esposito et al., 2011), anxiety- and mood-related disorders (Campos et al., 2013; Fogaça et al., 2018), as well as drug addiction (Luján et al., 2019).

Neurodegenerative and ischemic conditions are among the circumstances in which hippocampal function can manifest greater impairments (Shah et al., 2019). It was Esposito et al. (2011) who firstly reported the pro-neurogenic effect of CBD in a neuropathological disease model. In their work, they showed how CBD could restore the neuronal differentiation levels after β amyloid peptide inoculation in a rat model of Alzheimer’s disease. This effect was shown dependent on the activation of PPARγ receptors. Significantly, when a PPARγ antagonist was co-administered, CBD did not induce neuronal differentiation and, consequently, its neuroprotective effects were prevented (Esposito et al., 2011). Although suggestive, these results will need to be further verified, given the alternative protective consequences of PPARγ receptor activity by itself (Hughes and Herron, 2019). Anxiety- and mood-related disorders symptomatology is also critically dependent on hippocampal function (Anacker and Hen, 2017). For this reason, Campos et al. (2013) tested if the action of CBD on hippocampal neurogenesis accounted for its anxiolytic and antidepressant effects. Using a genetic-pharmacological approach, they were able to report that the blockade of adult neurogenesis accounted for the anxiolytic and antidepressant effects of CBD on the elevated plus-maze and novel suppressed feeding tests. Using a more indirect approach, Fogaça et al. (2018) have recently supported these implications. The co-administration of CBD and a CB1 or CB2 antagonist prevented both the increase in adult hippocampal neurogenesis and the anxiolytic effects of CBD. Again, this pharmacological strategy does not allow to discard beneficial changes induced by CB1 or CB2 receptor activity by itself, but the replication of the causal discovery of Campos et al. (2013) is certainly meaningful. Finally, our group also tried to unravel the participation of adult neurogenesis in the protective actions of CBD on cocaine self-administration, a rodent model of cocaine abuse. Based on the findings that CBD-induced attenuation of cocaine voluntary intake was accompanied by increased adult neurogenesis, as well as MAPK/CREB pathway activity in the hippocampus (Luján et al., 2018), we developed a pharmacological strategy aimed to prevent the increases in adult neurogenesis induced by CBD with the anti-mitotic agent temozolomide (Niibori et al., 2012). Similar to Campos et al. (2013), we found that such an increase was crucially required by CBD to reduce cocaine voluntary intake in mice (Luján et al., 2019). Overall, available data supports that CBD-induced adult neurogenesis can account for the protective effects of CBD in certain psychiatric conditions. The role of CBD neurogenesis in other mental diseases remains largely unexplored. The case of schizophrenia is especially suggesting. Decreased hippocampal neurogenesis is observed in schizophrenic patients compared with control subjects (Reif et al., 2006), and it is rescued by atypical antipsychotics in rodents (Kusumi et al., 2015). Noteworthy, the pro-neurogenic effects of CBD in mice exposed to chronic unpredictable stress suggestively resembles that of atypical antipsychotics such as clozapine in the same model (Campos et al., 2013; Chikama et al., 2017; Morais et al., 2017; Fogaça et al., 2018). Based on this observation, studies dissecting the importance of CBD pro-neurogenic effects on its antipsychotic properties are promising, as well as highly needed.

## Conclusion and Future Directions

A significant amount of animal and human data has emerged relating the neuro-modulatory role of adult hippocampal neurogenesis, its interactions with broader hippocampal circuits, and its implications on altered behaviors in different neuropsychiatric disorders (Beckervordersandforth and Rolando, 2020). Meanwhile, some pro-neurogenic compounds have been experimentally employed to counteract maladaptive neuroplasticity and improve hippocampal function. In the last decade, there has been an increased interest in the psychiatric therapeutic potential of CBD. Its protective brain effects, as well as its endocannabinoid mechanisms, have been related to its ability to facilitate the survival and differentiation of newborn neurons of the DG. Crucially, key studies have emerged linking this pro-neurogenic effect with reduced anxiety-like states and improved emotional and motivational processing in animal models of stress-, mood-, and substance use-related disorders. Albeit convincing, investigations of CBD’s pro-neurogenic effects are still in an early stage, and further experimental efforts are required to answer several open questions. Only two studies have so far fully addressed the causal implication of such a CBD mechanism (Campos et al., 2013; Luján et al., 2019). This lack of studies also leads to several replication needs. For example, most of the work has been developed in male mice, which hinders possible interpretations regarding sex- or species-specific effects. Also, evidence regarding the effects of CBD in the pre-mitotic stages of neuronal proliferation is scarce. On the other end, we still lack a direct electrophysiological confirmation of the functional integration of maturing neurons in conditions of elevated neurogenic state induced by CBD. Answering such a question is vital to clarify the functional relevance of CBD-induced neurogenesis and rule out an epiphenomenon effect. From a theoretical perspective, we also needed to better conceptualize the therapeutic potential of increased neurogenic states in adults. Newborn neurons necessarily remodel hippocampal circuitries upon functional integration. Thus, increased neurogenesis can destabilize consolidated memories (Chambers et al., 2004; Deisseroth et al., 2004), which may promote forgetting (Akers et al., 2014; but see Epp et al., 2016). Finally, indirect information supportive of the occurrence of hippocampal neurogenesis in humans treated with CBD is not yet available. Measures of the 1.28 ppm neurogenesis-specific peak using magnetic resonance spectroscopy (Manganas et al., 2007) could be incorporated in future clinical trials working with CBD treatments to shed more light on the functional and therapeutic relevance of these CBD’s neurogenic changes.

## Author Contributions

ML and OV were responsible for the study concept and design. Both authors drafted the manuscript and approved the final version for publication.

## Conflict of Interest

The authors declare that the research was conducted in the absence of any commercial or financial relationships that could be construed as a potential conflict of interest.

## References

[B1] Aguado T. Monory K. Palazuelos J. Stella‡ N. Cravatt B. Lutz B.. (2005). The endocannabinoid system drives neural progenitor proliferation. FASEB J. 19, 1704–1706. 10.1096/fj.05-3995fje 16037095

[B2] Aguado T. Romero E. Monory K. Palazuelos J. Sendtner M. Marsicano G.. (2007). The CB1 cannabinoid receptor mediates excitotoxicity-induced neural progenitor proliferation and neurogenesis. J. Biol. Chem. 282, 23892–23898. 10.1074/jbc.M700678200 17556369

[B3] Akers K. G. Martinez-Canabal A. Restivo L. Yiu A. P. De Cristofaro A. Hsiang H.-L.. (2014). Hippocampal neurogenesis regulates forgetting during adulthood and infancy. Science 344, 598–602. 10.1126/science.1248903 24812394

[B4] Altman J. Das G. D. (1965). Autoradiographic and histological evidence of postnatal hippocampal neurogenesis in rats. J. Comp. Neurol. 124, 319–335. 10.1002/cne.901240303 5861717

[B5] Amrein I. (2015). Adult hippocampal neurogenesis in natural populations of mammals. Cold Spring Harb. Perspect. Biol. 7:a021295. 10.1101/cshperspect.a021295 25934014 PMC4448614

[B6] Anacker C. Hen R. (2017). Adult hippocampal neurogenesis and cognitive flexibility—linking memory and mood. Nat. Rev. Neurosci. 18, 335–346. 10.1038/nrn.2017.45 28469276 PMC6261347

[B7] Andres-Mach M. Zagaja M. Haratym-Maj A. Rola R. Maj M. Haratym J.. (2017). A long-term treatment with arachidonyl-2^′^-chloroethylamide combined with valproate increases neurogenesis in a mouse pilocarpine model of epilepsy. Int. J. Mol. Sci. 18:900. 10.3390/ijms18050900 28441341 PMC5454813

[B8] Atalay S. Jarocka-Karpowicz I. Skrzydlewska E. (2019). Antioxidative and anti-inflammatory properties of cannabidiol. Antioxidants 9:21. 10.3390/antiox9010021 31881765 PMC7023045

[B9] Avraham H. K. Jiang S. Fu Y. Rockenstein E. Makriyannis A. Zvonok A.. (2014). The cannabinoid CB2 receptor agonist AM1241 enhances neurogenesis in GFAP/Gp120 transgenic mice displaying deficits in neurogenesis. Br. J. Pharmacol. 171, 468–479. 10.1111/bph.12478 24148086 PMC3904265

[B10] Bagot R. C. Parise E. M. Peña C. J. Zhang H.-X. Maze I. Chaudhury D.. (2015). Ventral hippocampal afferents to the nucleus accumbens regulate susceptibility to depression. Nat. Commun. 6:7062. 10.1038/ncomms8062 25952660 PMC4430111

[B11] Barr J. L. Bray B. Forster G. L. (2018). “The hippocampus as a neural link between negative affect and vulnerability for psychostimulant relapse,” in The Hippocampus—Plasticity and Functions, ed. Stuchlik A. (London, UK: IntechOpen Limited), 127–167.

[B12] Bazelot M. Bocchio M. Kasugai Y. Fischer D. Dodson P. D. Ferraguti F.. (2015). Hippocampal theta input to the amygdala shapes feedforward inhibition to gate heterosynaptic plasticity. Neuron 87, 1290–1303. 10.1016/j.neuron.2015.08.024 26402610 PMC4590554

[B13] Beckervordersandforth R. Rolando C. (2020). Untangling human neurogenesis to understand counteract brain disorders. Curr. Opin. Pharmacol. 50, 67–73. 10.1016/j.coph.2019.12.002 31901615

[B14] Bi G. Galaj E. He Y. Xi Z. (2019). Cannabidiol inhibits sucrose self-administration by CB1 and CB2 receptor mechanisms in rodents. Addict. Biol. [Epub ahead of print]. 10.1111/adb.12783 31215752 PMC6920611

[B15] Bis-Humbert C. García-Cabrerizo R. García-Fuster M. J. (2020). Decreased sensitivity in adolescent versus adult rats to the antidepressant-like effects of cannabidiol. Psychopharmacology 237, 1621–1631. 10.1007/s00213-020-05481-4 32086540

[B16] Bisogno T. Hanus L. De Petrocellis L. Tchilibon S. Ponde D. E. Brandi I.. (2001). Molecular targets for cannabidiol and its synthetic analogues: effect on vanilloid VR1 receptors and on the cellular uptake and enzymatic hydrolysis of anandamide. Br. J. Pharmacol. 134, 845–852. 10.1038/sj.bjp.0704327 11606325 PMC1573017

[B17] Boldrini M. Fulmore C. A. Tartt A. N. Simeon L. R. Pavlova I. Poposka V.. (2018). Human hippocampal neurogenesis persists throughout aging. Cell Stem Cell 22, 589.e5–599.e5. 10.1016/j.stem.2018.03.015 29625071 PMC5957089

[B18] Brandt M. D. Jessberger S. Steiner B. Kronenberg G. Reuter K. Bick-Sander A.. (2003). Transient calretinin expression defines early postmitotic step of neuronal differentiation in adult hippocampal neurogenesis of mice. Mol. Cell. Neurosci. 24, 603–613. 10.1016/s1044-7431(03)00207-0 14664811

[B19] Britt J. P. Benaliouad F. McDevitt R. A. Stuber G. D. Wise R. A. Bonci A. (2012). Synaptic and behavioral profile of multiple glutamatergic inputs to the nucleus accumbens. Neuron 76, 790–803. 10.1016/j.neuron.2012.09.040 23177963 PMC3607383

[B20] Calpe-López C. García-Pardo M. P. Aguilar M. A. (2019). Cannabidiol treatment might promote resilience to cocaine and methamphetamine use disorders: a review of possible mechanisms. Molecules 24:2583. 10.3390/molecules24142583 31315244 PMC6680550

[B23] Campos A. C. Fogaça M. V. Sonego A. B. Guimarães F. S. (2016). Cannabidiol, neuroprotection and neuropsychiatric disorders. Pharmacol. Res. 112, 119–127. 10.1016/j.phrs.2016.01.033 26845349

[B21] Campos A. C. Guimarães F. S. (2009). Evidence for a potential role for TRPV1 receptors in the dorsolateral periaqueductal gray in the attenuation of the anxiolytic effects of cannabinoids. Prog. Neuropsychopharmacol. Biol. Psychiatry 33, 1517–1521. 10.1016/j.pnpbp.2009.08.017 19735690

[B1100] Campos A. C. Fogaça M. V. Scarante F. F. Joca S. R. L. Sales A. J. Gomes F. V.. (2017). Plastic and neuroprotective mechanisms involved in the therapeutic effects of cannabidiol in psychiatric disorders. Front. Pharmacol. 8:269. 10.3389/fphar.2017.00269 28588483 PMC5441138

[B24] Campos A. C. Ortega Z. Palazuelos J. Fogaça M. V. Aguiar D. C. Díaz-Alonso J.. (2013). The anxiolytic effect of cannabidiol on chronically stressed mice depends on hippocampal neurogenesis: involvement of the endocannabinoid system. Int. J. Neuropsychopharmacol. 16, 1407–1419. 10.1017/s1461145712001502 23298518

[B25] Castilla-Ortega E. Serrano A. Blanco E. Araos P. Suárez J. Pavón F. J.. (2016). A place for the hippocampus in the cocaine addiction circuit: potential roles for adult hippocampal neurogenesis. Neurosci. Biobehav. Rev. 66, 15–32. 10.1016/j.neubiorev.2016.03.030 27118134

[B26] Chambers R. A. (2013). Adult hippocampal neurogenesis in the pathogenesis of addiction and dual diagnosis disorders. Drug Alcohol Depend. 130, 1–12. 10.1016/j.drugalcdep.2012.12.005 23279925 PMC3640791

[B27] Chambers R. A. Potenza M. N. Hoffman R. E. Miranker W. (2004). Simulated apoptosis/neurogenesis regulates learning and memory capabilities of adaptive neural networks. Neuropsychopharmacology 29, 747–758. 10.1038/sj.npp.1300358 14702022

[B28] Chambers R. Self W. (2002). Motivational responses to natural and drug rewards in rats with neonatal ventral hippocampal lesions an animal model of dual diagnosis schizophrenia. Neuropsychopharmacology 27, 889–905. 10.1016/s0893-133x(02)00365-2 12464446 PMC2919158

[B29] Chesney E. Oliver D. Green A. Sovi S. Wilson J. Englund A.. (2020). Adverse effects of cannabidiol: a systematic review and meta-analysis of randomized clinical trials. Neuropsychopharmacology [Epub ahead of print]. 10.1038/s41386-020-0667-2 32268347 PMC7608221

[B30] Chikama K. Yamada H. Tsukamoto T. Kajitani K. Nakabeppu Y. Uchimura N. (2017). Chronic atypical antipsychotics, but not haloperidol, increase neurogenesis in the hippocampus of adult mouse. Brain Res. 1676, 77–82. 10.1016/j.brainres.2017.09.006 28899760

[B31] Cuthbert B. N. (2014). The RDoC framework: facilitating transition from ICD/DSM to dimensional approaches that integrate neuroscience and psychopathology. World Psychiatry 13, 28–35. 10.1002/wps.20087 24497240 PMC3918011

[B33] Deisseroth K. Singla S. Toda H. Monje M. Palmer T. D. Malenka R. C. (2004). Excitation-neurogenesis coupling in adult neural stem/progenitor cells. Neuron 42, 535–552. 10.1016/s0896-6273(04)00266-1 15157417

[B34] Demirakca T. Sartorius A. Ende G. Meyer N. Welzel H. Skopp G.. (2010). Diminished gray matter in the hippocampus of cannabis users: possible protective effects of cannabidiol. Drug Alcohol Depend. 114, 242–245. 10.1016/j.drugalcdep.2010.09.020 21050680

[B32] De Petrocellis L. Ligresti A. Moriello A. S. Allarà M. Bisogno T. Petrosino S.. (2011). Effects of cannabinoids and cannabinoid-enriched Cannabis extracts on TRP channels and endocannabinoid metabolic enzymes. Br. J. Pharmacol. 163, 1479–1494. 10.1111/j.1476-5381.2010.01166.x 21175579 PMC3165957

[B35] Deroche-Gamonet V. Revest J. M. Fiancette J. F. Balado E. Koehl M. Grosjean N.. (2019). Depleting adult dentate gyrus neurogenesis increases cocaine-seeking behavior. Mol. Psychiatry 24, 312–320. 10.1038/s41380-018-0038-0 29507372

[B36] Dhanabalan G. Le Maître T. W. Bogdanovic N. Alkass K. Druid H. (2018). Hippocampal granule cell loss in human chronic alcohol abusers. Neurobiol. Dis. 120, 63–75. 10.1016/j.nbd.2018.08.011 30189262

[B37] Drew L. J. Kheirbek M. A. Luna V. M. Denny C. A. Cloidt M. A. Wu M. V.. (2016). Activation of local inhibitory circuits in the dentate gyrus by adult-born neurons. Hippocampus 26, 763–778. 10.1002/hipo.22557 26662922 PMC4867135

[B38] Elsaid S. Le Foll B. (2020). The complexity of pharmacology of cannabidiol (CBD) and its implications in the treatment of brain disorders. Neuropsychopharmacology 45, 229–230. 10.1038/s41386-019-0518-1 31511618 PMC6879582

[B39] Encinas J. M. Vaahtokari A. Enikolopov G. (2006). Fluoxetine targets early progenitor cells in the adult brain. Proc. Natl. Acad. Sci. U S A 103, 8233–8238. 10.1073/pnas.0601992103 16702546 PMC1461404

[B40] Epp J. R. Silva Mera R. Köhler S. Josselyn S. A. Frankland P. W. (2016). Neurogenesis-mediated forgetting minimizes proactive interference. Nat. Commun. 7:10838. 10.1038/ncomms10838 26917323 PMC4773435

[B41] Esposito G. Scuderi C. Valenza M. Togna G. I. Latina V. De Filippis D.. (2011). Cannabidiol reduces Aβ-induced neuroinflammation and promotes hippocampal neurogenesis through PPARγ involvement. PLoS One 6:e28668. 10.1371/journal.pone.0028668 22163051 PMC3230631

[B42] Felix-Ortiz A. C. Beyeler A. Seo C. Leppla C. A. Wildes C. P. Tye K. M. (2013). BLA to vHPC inputs modulate anxiety-related behaviors. Neuron 79, 658–664. 10.1016/j.neuron.2013.06.016 23972595 PMC4205569

[B43] Fogaça M. V. Campos A. C. Coelho L. D. Duman R. S. Guimarães F. S. (2018). The anxiolytic effects of cannabidiol in chronically stressed mice are mediated by the endocannabinoid system: role of neurogenesis and dendritic remodeling. Neuropharmacology 135, 22–33. 10.1016/j.neuropharm.2018.03.001 29510186

[B44] Galaj E. Bi G.-H. Yang H.-J. Xi Z.-X. (2020). Cannabidiol attenuates the rewarding effects of cocaine in rats by CB2, 5-HT_1A_ and TRPV1 receptor mechanisms. Neuropharmacology 167:107740. 10.1016/j.neuropharm.2019.107740 31437433 PMC7493134

[B45] Ge S. Yang C.-H. Hsu K.-S. Ming G.-L. Song H. (2007). A critical period for enhanced synaptic plasticity in newly generated neurons of the adult brain. Neuron 54, 559–566. 10.1016/j.neuron.2007.05.002 17521569 PMC2040308

[B46] Giacoppo S. Pollastro F. Grassi G. Bramanti P. Mazzon E. (2017). Target regulation of PI3K/Akt/mTOR pathway by cannabidiol in treatment of experimental multiple sclerosis. Fitoterapia 116, 77–84. 10.1016/j.fitote.2016.11.010 27890794

[B47] Gonzalez-Cuevas G. Martin-Fardon R. Kerr T. M. Stouffer D. G. Parsons L. H. Hammell D. C.. (2018). Unique treatment potential of cannabidiol for the prevention of relapse to drug use: preclinical proof of principle. Neuropsychopharmacology 43, 2036–2045. 10.1038/s41386-018-0050-8 29686308 PMC6098033

[B48] Hill J. D. Zuluaga-Ramirez V. Gajghate S. Winfield M. Sriram U. Rom S.. (2019). Activation of GPR55 induces neuroprotection of hippocampal neurogenesis and immune responses of neural stem cells following chronic, systemic inflammation. Brain. Behav. Immun. 76, 165–181. 10.1016/j.bbi.2018.11.017 30465881 PMC6398994

[B49] Hughes B. Herron C. E. (2019). Cannabidiol reverses deficits in hippocampal LTP in a model of Alzheimer’s disease. Neurochem. Res. 44, 703–713. 10.1007/s11064-018-2513-z 29574668

[B50] Ikrar T. Guo N. He K. Besnard A. Levinson S. Hill A.. (2013). Adult neurogenesis modifies excitability of the dentate gyrus. Front. Neural Circuits 7:204. 10.3389/fncir.2013.00204 24421758 PMC3872742

[B51] Jurkus R. Day H. L. L. Guimarães F. S. Lee J. L. C. Bertoglio L. J. Stevenson C. W. (2016). Cannabidiol regulation of learned fear: implications for treating anxiety-related disorders. Front. Pharmacol. 7:454. 10.3389/fphar.2016.00454 27932983 PMC5121237

[B52] Katsidoni V. Anagnostou I. Panagis G. (2013). Cannabidiol inhibits the reward-facilitating effect of morphine: involvement of 5-HT_1A_ receptors in the dorsal raphe nucleus. Addict. Biol. 18, 286–296. 10.1111/j.1369-1600.2012.00483.x 22862835

[B53] Kempermann G. (2012). New neurons for “survival of the fittest”. Nat. Rev. Neurosci. 13, 727–736. 10.1038/nrn3319 22948073

[B54] Kempermann G. Song H. Gage F. H. (2015). Neurogenesis in the adult hippocampus. Cold Spring Harb. Perspect. Biol. 7:a018929. 10.1101/cshperspect.a018929 26330519 PMC4563705

[B55] Koob G. F. (2015). The dark side of emotion: the addiction perspective. Eur. J. Pharmacol. 753, 73–787. 10.1016/j.ejphar.2014.11.044 25583178 PMC4380644

[B701] Kriegstein A. Alvarez-Buylla A. (2009). The glial nature of embryonic and adult neural stem cells. Annu. Rev. Neurosci. 32, 149–184. 10.1146/annurev.neuro.051508.135600 19555289 PMC3086722

[B56] Kuhn H. G. Toda T. Gage F. H. (2018). Adult hippocampal neurogenesis: a coming-of-age story. J. Neurosci. 38, 10401–10410. 10.1523/jneurosci.2144-18.2018 30381404 PMC6284110

[B57] Kusumi I. Boku S. Takahashi Y. (2015). Psychopharmacology of atypical antipsychotic drugs: from the receptor binding profile to neuroprotection and neurogenesis. Psychiatry Clin. Neurosci. 69, 243–258. 10.1111/pcn.12242 25296946

[B58] Ladrón de Guevara-Miranda D. Moreno-Fernández R. D. Gil-Rodríguez S. Rosell-Valle C. Estivill-Torrús G. Serrano A.. (2019). Lysophosphatidic acid-induced increase in adult hippocampal neurogenesis facilitates the forgetting of cocaine-contextual memory. Addict. Biol. 24, 458–470. 10.1111/adb.12612 29480526

[B59] Lanza Cariccio V. Scionti D. Raffa A. Iori R. Pollastro F. Diomede F.. (2018). Treatment of periodontal ligament stem cells with MOR and CBD promotes cell survival and neuronal differentiation *via* the PI3K/Akt/mTOR pathway. Int. J. Mol. Sci. 19:2341. 10.3390/ijms19082341 30096889 PMC6121255

[B60] Laprairie R. B. Bagher A. M. Kelly M. E. M. Denovan-Wright E. M. (2015). Cannabidiol is a negative allosteric modulator of the cannabinoid CB1 receptor. Br. J. Pharmacol. 172, 4790–4805. 10.1111/bph.13250 26218440 PMC4621983

[B61] Leutgeb J. K. Leutgeb S. Moser M.-B. Moser E. I. (2007). Pattern separation in the dentate gyrus and CA3 of the hippocampus. Science 315, 961–966. 10.1126/science.1135801 17303747

[B62] Leweke F. M. Piomelli D. Pahlisch F. Muhl D. Gerth C. W. Hoyer C.. (2012). Cannabidiol enhances anandamide signaling and alleviates psychotic symptoms of schizophrenia. Transl. Psychiatry 2:e94. 10.1038/tp.2012.15 22832859 PMC3316151

[B63] Ligresti A. De Petrocellis L. Di Marzo V. (2016). From phytocannabinoids to cannabinoid receptors and endocannabinoids: pleiotropic physiological and pathological roles through complex pharmacology. Physiol. Rev. 96, 1593–1659. 10.1152/physrev.00002.2016 27630175

[B64] Linge R. Jiménez-Sánchez L. Campa L. Pilar-Cuéllar F. Vidal R. Pazos A.. (2016). Cannabidiol induces rapid-acting antidepressant-like effects and enhances cortical 5-HT/glutamate neurotransmission: role of 5-HT1A receptors. Neuropharmacology 103, 16–26. 10.1016/j.neuropharm.2015.12.017 26711860

[B66] Lucassen P. J. Stumpel M. W. Wang Q. Aronica E. (2010). Decreased numbers of progenitor cells but no response to antidepressant drugs in the hippocampus of elderly depressed patients. Neuropharmacology 58, 940–949. 10.1016/j.neuropharm.2010.01.012 20138063

[B67] Lujan M. A. Alegre-Zurano L. Martin-Sanchez A. Valverde O. (2020). The effects of cannabidiol on cue- and stress-induced reinstatement of cocaine seeking behavior in mice are reverted by the CB1 receptor antagonist AM4113. bioRxiv Neurosci. 10.1101/2020.01.23.916601 34688811

[B68] Luján M. Á. Cantacorps L. Valverde O. (2019). The pharmacological reduction of hippocampal neurogenesis attenuates the protective effects of cannabidiol on cocaine voluntary intake. Addict. Biol. 4:e12778. 10.1111/adb.12778 31162770

[B69] Luján M. Á. Castro-Zavala A. Alegre-Zurano L. Valverde O. (2018). Repeated Cannabidiol treatment reduces cocaine intake and modulates neural proliferation and CB1R expression in the mouse hippocampus. Neuropharmacology 143, 163–175. 10.1016/j.neuropharm.2018.09.043 30273593

[B70] Mandyam C. D. Koob G. F. (2012). The addicted brain craves new neurons: putative role for adult-born progenitors in promoting recovery. Trends Neurosci. 35, 250–260. 10.1016/j.tins.2011.12.005 22265158 PMC3321119

[B71] Manganas L. N. Zhang X. Li Y. Hazel R. D. Smith S. D. Wagshul M. E.. (2007). Magnetic resonance spectroscopy identifies neural progenitor cells in the live human brain. Science 318, 980–985. 10.1126/science.1147851 17991865 PMC4039561

[B72] Martínez-Pinilla E. Varani K. Reyes-Resina I. Angelats E. Vincenzi F. Ferreiro-Vera C.. (2017). Binding and signaling studies disclose a potential allosteric site for cannabidiol in cannabinoid CB2 receptors. Front. Pharmacol. 8:744. 10.3389/fphar.2017.00744 29109685 PMC5660261

[B73] McPartland J. M. Duncan M. Di Marzo V. Pertwee R. G. (2015). Are cannabidiol and ^Δ^9-tetrahydrocannabivarin negative modulators of the endocannabinoid system? A systematic review. Br. J. Pharmacol. 172, 737–753. 10.1111/bph.12944 25257544 PMC4301686

[B74] Mensching L. Djogo N. Keller C. Rading S. Karsak M. (2019). Stable adult hippocampal neurogenesis in cannabinoid receptor CB2 deficient mice. Int. J. Mol. Sci. 20:3759. 10.3390/ijms20153759 31374821 PMC6696320

[B75] Morais M. Patrício P. Mateus-Pinheiro A. Alves N. D. MacHado-Santos A. R. Correia J. S.. (2017). The modulation of adult neuroplasticity is involved in the mood-improving actions of atypical antipsychotics in an animal model of depression. Transl. Psychiatry 7:e1146. 10.1038/tp.2017.120 28585931 PMC5537642

[B76] Moreno-Jiménez E. P. Flor-García M. Terreros-Roncal J. Rábano A. Cafini F. Pallas-Bazarra N.. (2019). Adult hippocampal neurogenesis is abundant in neurologically healthy subjects and drops sharply in patients with Alzheimer’s disease. Nat. Med. 25, 554–560. 10.1038/s41591-019-0375-9 30911133

[B77] Mori M. A. Meyer E. Soares L. M. Milani H. Guimarães F. S. de Oliveira R. M. W. (2017). Cannabidiol reduces neuroinflammation and promotes neuroplasticity and functional recovery after brain ischemia. Prog. Neuropsychopharmacol. Biol. Psychiatry 75, 94–105. 10.1016/j.pnpbp.2016.11.005 27889412

[B78] Navarro G. Reyes-Resina I. Rivas-Santisteban R. Sánchez de Medina V. Morales P. Casano S.. (2018). Cannabidiol skews biased agonism at cannabinoid CB1 and CB2 receptors with smaller effect in CB1-CB2 heteroreceptor complexes. Biochem. Pharmacol. 157, 148–158. 10.1016/j.bcp.2018.08.046 30194918

[B79] Niibori Y. Yu T.-S. Epp J. R. Akers K. G. Josselyn S. A. Frankland P. W. (2012). Suppression of adult neurogenesis impairs population coding of similar contexts in hippocampal CA3 region. Nat. Commun. 3:1253. 10.1038/ncomms2261 23212382 PMC4931925

[B80] Noonan M. A. Bulin S. E. Fuller D. C. Eisch A. J. (2010). Reduction of adult hippocampal neurogenesis confers vulnerability in an animal model of cocaine addiction. J. Neurosci. 30, 304–315. 10.1523/jneurosci.4256-09.2010 20053911 PMC2844797

[B81] Norris C. Loureiro M. Kramar C. Zunder J. Renard J. Rushlow W.. (2016). Cannabidiol modulates fear memory formation through interactions with serotonergic transmission in the mesolimbic system. Neuropsychopharmacology 41, 2839–2850. 10.1038/npp.2016.93 27296152 PMC5061893

[B82] Oddi S. Scipioni L. Maccarrone M. (2020). Endocannabinoid system and adult neurogenesis: a focused review. Curr. Opin. Pharmacol. 50, 25–32. 10.1016/j.coph.2019.11.002 31864101

[B83] Osborne A. L. Solowij N. Weston-Green K. (2017). A systematic review of the effect of cannabidiol on cognitive function: relevance to schizophrenia. Neurosci. Biobehav. Rev. 72, 310–324. 10.1016/j.neubiorev.2016.11.012 27884751

[B84] Padilla-Coreano N. Bolkan S. S. Pierce G. M. Blackman D. R. Hardin W. D. Garcia-Garcia A. L.. (2016). Direct ventral hippocampal-prefrontal input is required for anxiety-related neural activity and behavior. Neuron 89, 857–866. 10.1016/j.neuron.2016.01.011 26853301 PMC4760847

[B85] Palazuelos J. Ortega Z. Díaz-Alonso J. Guzmán M. Galve-Roperh I. (2012). CB2 cannabinoid receptors promote neural progenitor cell proliferation *via* mTORC1 signaling. J. Biol. Chem. 287, 1198–1209. 10.1074/jbc.m111.291294 22102284 PMC3256884

[B86] Petrosino S. Verde R. Vaia M. Allarà M. Iuvone T. Di Marzo V. (2018). Anti-inflammatory properties of cannabidiol, a nonpsychotropic cannabinoid, in experimental allergic contact dermatitis. J. Pharmacol. Exp. Ther. 365, 652–663. 10.1124/jpet.117.244368 29632236

[B87] Prenderville J. A. Kelly Á. M. Downer E. J. (2015). The role of cannabinoids in adult neurogenesis. Br. J. Pharmacol. 172, 3950–3963. 10.1111/bph.13186 25951750 PMC4543605

[B88] Redondo R. L. Kim J. Arons A. L. Ramirez S. Liu X. Tonegawa S. (2014). Bidirectional switch of the valence associated with a hippocampal contextual memory engram. Nature 513, 426–430. 10.1038/nature13725 25162525 PMC4169316

[B89] Reif A. Fritzen S. Finger M. Strobel A. Lauer M. Schmitt A.. (2006). Neural stem cell proliferation is decreased in schizophrenia, but not in depression. Mol. Psychiatry 11, 514–522. 10.1038/sj.mp.4001791 16415915

[B90] Renard J. Loureiro M. Rosen L. G. Zunder J. de Oliveira C. Schmid S.. (2016). Cannabidiol counteracts amphetamine-induced neuronal and behavioral sensitization of the mesolimbic dopamine pathway through a novel mTOR/p70S6 kinase signaling pathway. J. Neurosci. 36, 5160–5169. 10.1523/jneurosci.3387-15.2016 27147666 PMC4854973

[B91] Renard J. Norris C. Rushlow W. Laviolette S. R. (2017). Neuronal and molecular effects of cannabidiol on the mesolimbic dopamine system: implications for novel schizophrenia treatments. Neurosci. Biobehav. Rev. 75, 157–165. 10.1016/j.neubiorev.2017.02.006 28185872

[B92] Revest J.-M. Dupret D. Koehl M. Funk-Reiter C. Grosjean N. Piazza P.-V.. (2009). Adult hippocampal neurogenesis is involved in anxiety-related behaviors. Mol. Psychiatry 14, 959–967. 10.1038/mp.2009.15 19255582

[B93] Rodrigues R. S. Ribeiro F. F. Ferreira F. Vaz S. H. Sebastião A. M. Xapelli S. (2017). Interaction between cannabinoid type 1 and type 2 receptors in the modulation of subventricular zone and dentate gyrus neurogenesis. Front. Pharmacol. 8:516. 10.3389/fphar.2017.00516 28848435 PMC5554396

[B94] Sahay A. Scobie K. N. Hill A. S. O’Carroll C. M. Kheirbek M. A. Burghardt N. S.. (2011). Increasing adult hippocampal neurogenesis is sufficient to improve pattern separation. Nature 472, 466–470. 10.1038/nature09817 21460835 PMC3084370

[B95] Sales A. J. Fogaça M. V. Sartim A. G. Pereira V. S. Wegener G. Guimarães F. S.. (2019). Cannabidiol induces rapid and sustained antidepressant-like effects through increased BDNF signaling and synaptogenesis in the prefrontal cortex. Mol. Neurobiol. 56, 1070–1081. 10.1007/s12035-018-1143-4 29869197

[B96] Santarelli L. Saxe M. Gross C. Surget A. Battaglia F. Dulawa S.. (2003). Requirement of hippocampal neurogenesis for the behavioral effects of antidepressants. Science 301, 805–809. 10.1126/science.1083328 12907793

[B97] Sartim A. G. Guimarães F. S. Joca S. R. L. (2016). Antidepressant-like effect of cannabidiol injection into the ventral medial prefrontal cortex—Possible involvement of 5-HT1A and CB1 receptors. Behav. Brain Res. 303, 218–227. 10.1016/j.bbr.2016.01.033 26801828

[B98] Sartim A. G. Sales A. J. Guimarães F. S. Joca S. R. L. (2018). Hippocampal mammalian target of rapamycin is implicated in stress-coping behavior induced by cannabidiol in the forced swim test. J. Psychopharmacol. 32, 922–931. 10.1177/0269881118784877 29968502

[B99] Schiavon A. P. Bonato J. M. Milani H. Guimarães F. S. Weffort de Oliveira R. M. (2016). Influence of single and repeated cannabidiol administration on emotional behavior and markers of cell proliferation and neurogenesis in non-stressed mice. Prog. Neuropsychopharmacol. Biol. Psychiatry 64, 27–34. 10.1016/j.pnpbp.2015.06.017 26187374

[B100] Sentir A. M. Bell R. L. Engleman E. A. Chambers R. A. (2020). Polysubstance addiction vulnerability in mental illness: concurrent alcohol and nicotine self-administration in the neurodevelopmental hippocampal lesion rat model of schizophrenia. Addict. Biol. 25:e12704. 10.1111/adb.12704 30592364

[B101] Shah F. A. Li T. Kury L. T. A. Zeb A. Khatoon S. Liu G.. (2019). Pathological comparisons of the hippocampal changes in the transient and permanent middle cerebral artery occlusion rat models. Front. Neurol. 10:1178. 10.3389/fneur.2019.01178 31798514 PMC6868119

[B102] Shinjyo N. Di Marzo V. (2013). The effect of cannabichromene on adult neural stem/progenitor cells. Neurochem. Int. 63, 432–437. 10.1016/j.neuint.2013.08.002 23941747

[B103] Silote G. P. Sartim A. Sales A. Eskelund A. Guimarães F. S. Wegener G.. (2019). Emerging evidence for the antidepressant effect of cannabidiol and the underlying molecular mechanisms. J. Chem. Neuroanat. 98, 104–116. 10.1016/j.jchemneu.2019.04.006 31039391

[B104] Snyder J. S. Soumier A. Brewer M. Pickel J. Cameron H. A. (2011). Adult hippocampal neurogenesis buffers stress responses and depressive behaviour. Nature 476, 458–461. 10.1038/nature10287 21814201 PMC3162077

[B105] Sorrells S. F. Paredes M. F. Cebrian-Silla A. Sandoval K. Qi D. Kelley K. W.. (2018). Human hippocampal neurogenesis drops sharply in children to undetectable levels in adults. Nature 555, 377–381. 10.1038/nature25975 29513649 PMC6179355

[B106] Spear L. P. (2018). Effects of adolescent alcohol consumption on the brain and behaviour. Nat. Rev. Neurosci. 19, 197–214. 10.1038/nrn.2018.10 29467469

[B107] Sun G. J. Sailor K. A. Mahmood Q. A. Chavali N. Christian K. M. Song H.. (2013). Seamless reconstruction of intact adult-born neurons by serial end-block imaging reveals complex axonal guidance and development in the adult hippocampus. J. Neurosci. 33, 11400–11411. 10.1523/jneurosci.1374-13.2013 23843512 PMC3724551

[B108] Taffe M. A. Kotzebue R. W. Crean R. D. Crawford E. F. Edwards S. Mandyam C. D. (2010). Long-lasting reduction in hippocampal neurogenesis by alcohol consumption in adolescent nonhuman primates. Proc. Natl. Acad. Sci. U S A 107, 11104–11109. 10.1073/pnas.0912810107 20534463 PMC2890755

[B109] Tello-Ramos M. C. Branch C. L. Kozlovsky D. Y. Pitera A. M. Pravosudov V. V. (2019). Spatial memory and cognitive flexibility trade-offs: to be or not to be flexible, that is the question. Anim. Behav. 147, 129–136. 10.1016/j.anbehav.2018.02.019

[B110] Temprana S. G. Mongiat L. A. Yang S. M. Trinchero M. F. Alvarez D. D. Kropff E.. (2015). Delayed coupling to feedback inhibition during a critical period for the integration of adult-born granule cells. Neuron 85, 116–130. 10.1016/j.neuron.2014.11.023 25533485 PMC4329739

[B111] Tham M. Yilmaz O. Alaverdashvili M. Kelly M. E. M. Denovan-Wright E. M. Laprairie R. B. (2019). Allosteric and orthosteric pharmacology of cannabidiol and cannabidiol-dimethylheptyl at the type 1 and type 2 cannabinoid receptors. Br. J. Pharmacol. 176, 1455–1469. 10.1111/bph.14440 29981240 PMC6487556

[B112] Tobin M. K. Musaraca K. Disouky A. Shetti A. Bheri A. Honer W. G.. (2019). Human hippocampal neurogenesis persists in aged adults and Alzheimer’s disease patients. Cell Stem Cell 24, 974.e3–982.e3. 10.1016/j.stem.2019.05.003 31130513 PMC6608595

[B113] Turner S. E. Williams C. M. Iversen L. Whalley B. J. (2017). Molecular pharmacology of phytocannabinoids. Prog. Chem. Org. Nat. Prod. 103, 61–101. 10.1007/978-3-319-45541-9_3 28120231

[B65] Volkow N. D. Koob G. F. McLellan A. T. (2016). Neurobiologic advances from the brain disease model of addiction. N. Engl. J. Med. 374, 363–371. 10.1056/nejmra1511480 26816013 PMC6135257

[B114] Waterhouse E. G. An J. J. Orefice L. L. Baydyuk M. Liao G.-Y. Zheng K.. (2012). BDNF promotes differentiation and maturation of adult-born neurons through GABAergic transmission. J. Neurosci. 32, 14318–14330. 10.1523/jneurosci.0709-12.2012 23055503 PMC3519245

[B115] Wolf S. A. Bick-Sander A. Fabel K. Leal-Galicia P. Tauber S. Ramirez-Rodriguez G.. (2010). Cannabinoid receptor CB1 mediates baseline and activity-induced survival of new neurons in adult hippocampal neurogenesis. Cell Commun. Signal. 8:12. 10.1186/1478-811x-8-12 20565726 PMC2898685

[B116] Wu M. V. Shamy J. L. Bedi G. Choi C.-W. J. Wall M. M. Arango V.. (2014). Impact of social status and antidepressant treatment on neurogenesis in the baboon hippocampus. Neuropsychopharmacology 39, 1861–1871. 10.1038/npp.2014.33 24518288 PMC4059894

[B117] Zimmermann T. Ludewig S. Korte M. Lutz B. Leschik J. (2016). Functional impact of the cannabinoid type 1 receptor in adult neurogenesis. Eur. Neuropsychopharmacol. 26, S186–S187. 10.1016/s0924-977x(16)31021-5

[B118] Zlebnik N. E. Cheer J. F. (2016). Beyond the CB1 receptor: is cannabidiol the answer for disorders of motivation? Annu. Rev. Neurosci. 39, 1–17. 10.1146/annurev-neuro-070815-014038 27023732 PMC5818147

